# Role of Glia in Memory Deficits Following Traumatic Brain Injury: Biomarkers of Glia Dysfunction

**DOI:** 10.3389/fnint.2016.00007

**Published:** 2016-02-29

**Authors:** Venkata S. S. S. Sajja, Nora Hlavac, Pamela J. VandeVord

**Affiliations:** ^1^Cellular Imaging Section and Vascular Biology Program, Department of Radiology and Radiological Science, Institute for Cell Engineering, Johns Hopkins University School of MedicineBaltimore, MA, USA; ^2^Department of Biomedical Engineering and Mechanics, Virginia Tech UniversityBlacksburg, VA, USA

**Keywords:** astrocytes, microglia, oligodendrocytes, traumtic brain injury (TBI), biomarkers, MRS spectroscopy, memory impairment, gliosis

## Abstract

Historically, glial cells have been recognized as a structural component of the brain. However, it has become clear that glial cells are intimately involved in the complexities of neural networks and memory formations. Astrocytes, microglia, and oligodendrocytes have dynamic responsibilities which substantially impact neuronal function and activities. Moreover, the importance of glia following brain injury has come to the forefront in discussions to improve axonal regeneration and functional recovery. The numerous activities of glia following injury can either promote recovery or underlie the pathobiology of memory deficits. This review outlines the pathological states of glial cells which evolve from their positive supporting roles to those which disrupt synaptic function and neuroplasticity following injury. Evidence suggests that glial cells interact extensively with neurons both chemically and physically, reinforcing their role as pivotal for higher brain functions such as learning and memory. Collectively, this mini review surveys investigations of how glial dysfunction following brain injury can alter mechanisms of synaptic plasticity and how this may be related to an increased risk for persistent memory deficits. We also include recent findings, that demonstrate new molecular avenues for clinical biomarker discovery.

## Introduction

It is generally accepted, that neurons make up less than 25% of the cells in the brain, yet are responsible for information processing and control of bodily functions. Astrocytes, which make up 30–65% of glia and are the most abundant cell type in the brain, are multifunctional cells whose roles include maintaining osmotic balance and optimal ionic conditions for neurons, information processing via neurotransmitter recycling, and metabolite homeostasis (Kimelberg, 2005; Buffo et al., 2010; Kimelberg and Nedergaard, 2010). Collectively, these functions, as well as others, make the astrocytes indirectly involved in all brain function including memory formation (Moraga-Amaro et al., 2014). Microglia compose approximately 10% of total glia in the brain and are mainly identified by their function as immune cells of the central nervous system (CNS), arriving first at the injury site to initiate the inflammatory cascade. However, evidence indicates that “resting” microglia play a critical role in regulating synaptic and structural plasticity during learning and memory (Kettenmann et al., 2011, 2013; Scheff et al., 2013). Lastly, oligodendrocytes provide support to axons with the production of the myelin sheath, which is vital for fast impulse conduction through the white matter (WM) tracts. These rapid interactions between brain regions are required for higher order brain functions like memory formation. Because of their high metabolic rates, oligodendrocytes are susceptible to the molecular consequences of tissue damage (McTigue and Tripathi, 2008). Oligodendrocyte death causes demyelination, impairment of axonal conduction, and ultimately axon death which contribute to memory impairment. Collectively, dysfunction of glia causes morphological and functional changes which effect the neural-glial and glial-glial interactions. Synaptic disconnections, imbalances of neurotransmitter homeostasis, and potential axonal degeneration and neuronal death can ultimately lead to memory impairment. Understanding the glia response, following injury at the molecular level may provide clues to decreasing chronic memory deficits.

## Secondary injury and metabolic dysfunction

**Figure 1:**
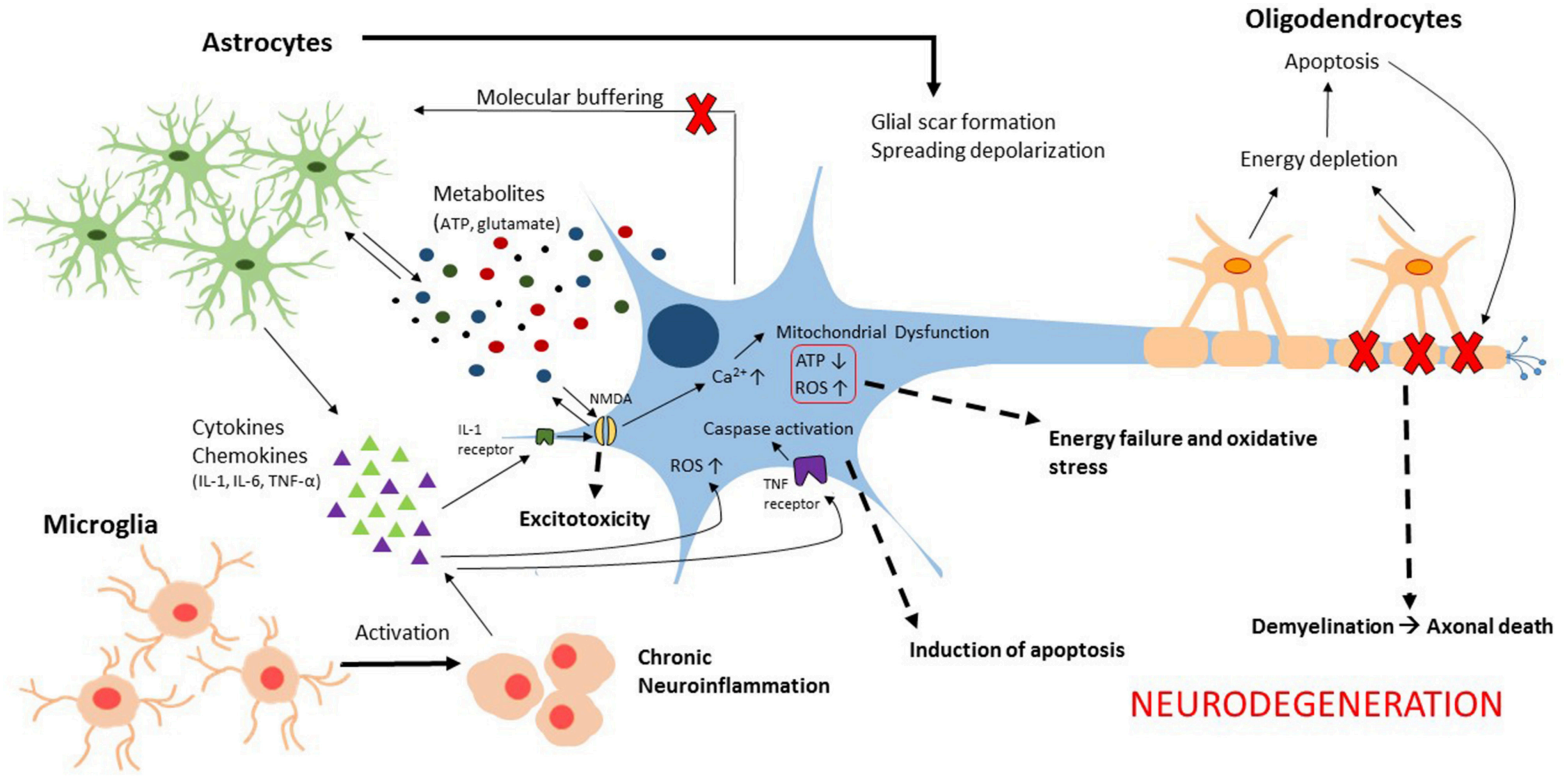
**Illustration of the glial contributions to secondary injury mechanisms associated with neurodegeneration following traumatic brain injury**.

Traumatic brain injury (TBI) is a complex, progressive condition that consists of primary and secondary injury mechanisms. Primary injury is due to direct mechanical insult and is the initiator of secondary molecular cascades. Secondary injury is characterized largely by metabolic imbalance and neuroinflammation (Figure 1). Following primary insult, brain cells experience energy depletion and a loss of calcium homeostasis, both of which are principal in mitochondrial function. Mitochondrial disruption is well documented in acute stages of TBI (Colicos and Dash, 1996; Xiong et al., 1997; Sullivan et al., 1998, 2005; Singh et al., 2006; Gilmer et al., 2009; Cheng et al., 2012). While these alterations are not glia-specific, they are intensified by activated glia. Because of the surge in extracellular ATP that results from damaged cells, glia are activated leading to downstream calcium release from endoplasmic reticulum (Locovei et al., 2006). Alterations in expression of various metabotropic receptors can occur as a result (Wang et al., 2012), contributing to surges of intracellular calcium. Increased cytosolic calcium is balanced by mitochondria at the expense of mitochondrial membrane potential. Eventually, mitochondria are driven to calcium overload and injury is exacerbated through generation of reactive oxygen species (ROS). Neurons are limited in their antioxidant capacity and thus rely on astrocytes to buffer ROS (Hamby and Sofroniew, 2010). Otherwise, they become susceptible to irreversible damage. Importantly, a pro-oxidative environment contributes to lipid, protein, and nucleic acid damage manifested largely in membrane disruption (Lewén and Hillered, 1998; Miller et al., 2015) and induction of neuroinflammation (Hsieh and Yang, 2013). Studies have concentrated on elucidating the roles of cellular sensors and enzymes that modulate intracellular calcium and ROS in metabolic dysfunction associated with death (Lu et al., 2014; Angeloni et al., 2015; Rao et al., 2015). Moreover, calcium signals in glial transmission are necessary for information processing and neuronal-glial coordination. Thus, impairment of glial-neuronal transmission contributes to memory loss (Walker and Tesco, 2013; Croft et al., 2015; Gundersen et al., 2015). In addition to calcium homeostasis, it is necessary to consider the consequence of potassium imbalances in secondary injury. Astrocytes normally uptake extracellular potassium via channels and Na+/K+/ATPase which in turn contributes to volume changes characteristic of TBI (Macaulay and Zeuthen, 2012; Larsen et al., 2014). Disruptions in potassium homeostasis, alongside neurotransmitter receptor activation, enhance neuronal impairment (D'Ambrosio et al., 1999; Pietrobon and Moskowitz, 2014).

## Trauma-associated edema

Cerebral edema is induced by water imbalance in response to trauma. Cytotoxic edema occurs in acute stages of TBI as a result of dysregulated metabolism. Often, there is a biphasic edema response in which early cytotoxic edema is followed by vasogenic edema associated with compromised blood-brain-barrier (BBB). Glia play an integral role in regulation of water and other molecules that transverse the BBB. Astrocytic endfeet directly contact brain vessels and are localized with aquaporin (AQP) proteins, which are pore proteins for water passage (Nielsen et al., 1997). Moreover, astrocytes are highly susceptible to swelling due to expression of AQPs (Suzuki et al., 2006; Satoh et al., 2007; Rao et al., 2011). Expression of AQP following trauma suggests that sustained AQP expression is critical in alleviating edema however, it is dependent on location relative to the injury, time, and variations in TBI models (Kiening et al., 2002; Sun et al., 2003; Zhao et al., 2005). A recent study reported only a small reduction in brain volume in AQP4 knockout mice with no evidence of difference in BBB disruption between AQP4 knockout and wildtype (Yao et al., 2015). Varied results may be related to a differential role of AQPs in the biphasic edema response. New theories hypothesize that AQP4 facilitates bulk flow through the glymphatic system, which poses contradiction to edema formation localized in astrocytic endfeet (Thrane et al., 2014). Evidence also suggests, that crosstalk exists between microglia and astrocytes in the regulation of AQP4 via microglial pattern recognition receptor-mediated pathways (Laird et al., 2014). Other studies are aimed to understand the effect of modulation of AQPs to ameliorate neuronal injury and cognitive deficits associated with TBI-induced edema (Tran et al., 2010; Shenaq et al., 2012).

## Extension of cellular death

Much of the intercellular molecular buffering required for homeostasis in the brain is mediated by gap junctions (GJs), which consist of connexin (Cx) hemichannels that transverse the plasma membrane directly connect adjacent cells. Cx30 and Cx43 are expressed by astrocytes while Cx32 is expressed only by oligodendrocytes (Rash et al., 2001). GJs are necessary for the formation of astrocytic networks that interconnect neurons synapses and vessels (Giaume, 2010; Giaume et al., 2010). The role of Cx43 in CNS injury has been debated as both protective and detrimental for GJ communications (Chew et al., 2010). GJs allow for the passage of ions, metabolites, and other small molecules. Thus, an injured cell can distribute its damaging components to adjacent healthy cells. While this is potentially protective for injured cells, it also exacerbates the spread of injury. Studies have investigated the role of Cx43 expression in the expansion of cellular death (Frantseva et al., 2002; Lin et al., 2008; Sun et al., 2014; Rovegno et al., 2015). Inflammatory cytokines secreted by microglia activate Cx43 in astrocytes and can enhance N-methyl-D-aspartate (NMDA) receptor-mediated excitotoxicity in surrounding neurons (Froger et al., 2010). Additionally, Cx hemichannels are a route for the release of ATP to extracellular space, which exacerbates metabolic dysfunction and inflammation (Cotrina et al., 1998; Frantseva et al., 2002; Davalos et al., 2005; Figiel et al., 2007). It is also known, that release of transmitters, including ATP and glutamate, can perturb intercellular calcium signaling within astrocytic networks, which in turn may contribute to neuroinflammation and cell death (Choo et al., 2013; De Bock et al., 2014). There is evidence that Cx expression influences functional and cognitive outcomes from injury (Huang et al., 2012; Sun et al., 2015) as well as progressive neurodegeneration (Orellana et al., 2009).

## Reactive gliosis

Subsequent to insult, glia are transformed into a reactive state. Reactive gliosis is characterized by specific molecular and morphologic changes in microglia and astrocytes. Upon activation, microglia in combination with macrophages and astrocytes secrete cytokines (interferon-γ, tissue necrosis factor-α, interleukins-1 and 6 as well as transforming growth factor-β (TGF-β)) (Morganti-Kossmann et al., 2001; Li et al., 2009; Kumar and Loane, 2012; Aungst et al., 2014; Sajja et al., 2014b). While activation is initiated immediately upon injury, it is often sustained chronically which is linked to damaging neuronal homeostasis and memory deficits (Hanisch and Kettenmann, 2007; Ramlackhansingh et al., 2011; Mannix and Whalen, 2012; Smith et al., 2012; Johnson et al., 2013). Neuroinflammation is associated with ROS and the exacerbation of astrocyte activation. Evidence of prolonged neurotrophic effects from activated microglia has been reported (Nagamoto-Combs et al., 2007). This chronic inflammation has detrimental effects and contributes to neurodegeneration and memory impairment (Faden and Loane, 2015). Approaches to molecular and genetic influence on decreased microglial activation have resulted in decreased neuropathology (Yi et al., 2008; Dohi et al., 2010) and improved cognitive and functional outcomes (Erlich et al., 2007; Li et al., 2009; Kabadi et al., 2012; Cho et al., 2013).

Astrocyte reactivity or astrogliosis, is characterized by three hallmarks: hypertrophy, increased expression of intermediate filaments (glial-fibrillary acidic protein (GFAP), nestin and vimentin), and increased proliferation (Baldwin and Scheff, 1996; Sahin Kaya et al., 1999; Vandevord et al., 2008). Astrogliosis is dependent on interplay with activated microglia (Di Giovanni et al., 2005; Myer et al., 2006). Reactive astrocytes secrete molecules for regulation of the existing neuroinflammatory response (Panenka et al., 2001; Gorina et al., 2011), are integral in creating physical barriers associated with the BBB, as well as contribute to scar formation around injured tissue. The astrocytic scar inhibits axonal regrowth as cells will secrete growth inhibitors, such as TGF-β, thus affecting long-term cognitive outcomes. Although, most research focuses on modulation of astrogliosis, both the protective and inhibitory effects have been evaluated in the context of improved neuronal survival and cognitive abilities over time (Smith et al., 1997; Hoane et al., 2003; Wu et al., 2010; Madathil et al., 2013).

## Glial contribution to memory deficits

Oligodendrocyte dysfunction due to inflammation or cellular death impairs neurotransmission via degeneration of WM tracts (Smith et al., 1997; Gorina et al., 2011). Pre-clinical and clinical studies have shown axonal disruption associated with functional impairment (Lu et al., 2012; MacDonald et al., 2013; Calabrese et al., 2014). A non-human primate study reported a loss of WM integrity and astrocytic hypertrophy with increased AQP-4 contributed to cell death associated with cognitive impairment (Lu et al., 2012). Specifically, learning and memory has been shown to be associated with abnormal levels of myo-inositol, which is an astrogliosis marker (Sajja et al., 2014b). Resultants of gliosis directed toward dementia, such as tau and DNA methylation markers are found to be upregulated following TBI (Bailey et al., 2015; Sajja et al., 2015; Shultz et al., 2015). Another indicator linked to memory deficits is the disrupted homeostasis of extra and intra-cellular K+ channels in glia (D'Ambrosio et al., 1999). Furthermore, it has shown, that by blocking glial activation, cognition was improved (Homsi et al., 2010; Bedi et al., 2013). New research has shown the role of ependymal cells in contributing to memory deficits. Ependymal cells are specialized glia, that line the ventricles of the CNS. Ependymal cell lose was found to decrease ventricular flow following TBI which could negatively affect the waste and nutrient exchange within the brain (Xiong et al., 2014). Additional research that helps decipher the molecular pathways between glia and memory deficits will be vital for development of better clinical tools for gauging memory loss.

## Glia-based biomarkers

**Figure 2:**
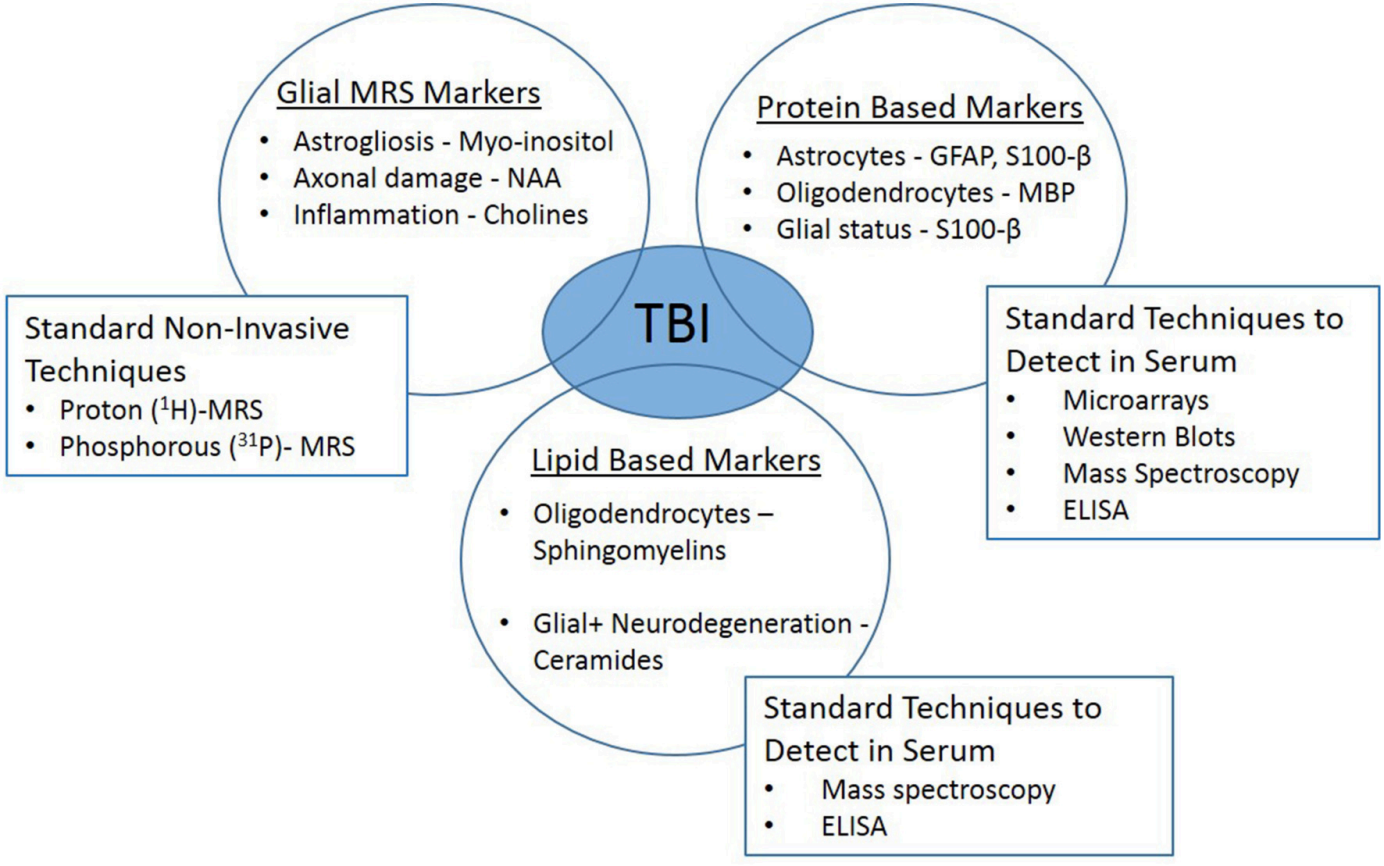
**Clinically translatable biomarkers for traumatic brain injury**. *In vivo* magnetic resonance spectroscopy (^1^H-MRS) and glial-specific serum biomarkers may provide the link needed to branch the basic and clinical research arenas.

The response of glia to TBI is multifaceted, supporting the importance of these cells to recovery. However, the intricate chemical and physical reactions of glia are very difficult to detect in the clinical setting. It is technically challenging to diagnosis and study the involvement of the glia in the recovery stages following injury and their contribution to memory deficits. Most minor TBI cases have normal findings in conventional neuroimaging [computed tomography (CT) and magnetic resonance imaging (MRI)]. While both basic and clinical research have made significant improvements, advancements are vital to fill the translational gap. Innovative technologies have emerged, such as serum biomarkers and *in vivo* magnetic resonance spectroscopy (^1^H-MRS) which may provide the link needed to branch the basic and clinical arenas (Figure 2).

### Serum biomarkers

Minimally invasive techniques, such as serum biomarkers, can be used to detect brain-specific pathologies. With technological advancements in proteomics and lipidomics, finding accurate biomarkers that reflect glial health status would be tremendously valuable. GFAP is a common astrocytic marker, that has been detected in serum following TBI in both pre-clinical and clinical studies (Fraser et al., 2011; Vajtr et al., 2012; Papa et al., 2014; Huang et al., 2015). Significant accumulation of GFAP persisted in blood up to 7 days post-injury (Svetlov et al., 2009; Boutte ´ et al., 2015). Some have suggested, that the use of GFAP as a TBI biomarker yields a net benefit above clinical screening alone and may help avoid costly imaging scans without sacrificing diagnostic sensitivity (McMahon et al., 2015). S100 calcium-binding protein B (S100-β) is another serum biomarker that is clinically used to help in diagnosis of neurological disorders (Bouvier et al., 2012; DeFazio et al., 2014; Thelin et al., 2014). S100-β is expressed primarily by mature astrocytes and is present in the extracellular space surrounding glia, assisting with regulation of the cell calcium influx/efflux, but also linked to apoptotic environments (Gyorgy et al., 2011; Vajtr et al., 2012). Studies have identified S100-β as biomarker that could potentially be used in TBI diagnosis, however, others suggest, that GFAP is a better evaluator of TBI without skull fractures (Papa et al., 2014). Myelin-basic protein (MBP) is a specific marker of oligodendrocytes and was detected in blood, indicating potential disruption in myelin, thus leading to axonal injury (Gyorgy et al., 2011; Yan et al., 2014; Papa et al., 2015).

Lipid-based biomarkers such as sphingolipids, specifically sphingomyelins and ceramides, have recently become an active area of biomarker research. Sphingomyelin is abundant in the myelin membrane and abnormal levels in blood can constitute changes in myelin health and associations with oligodendrocyte injury (Haughey, 2010; Abdullah et al., 2014; Novgorodov et al., 2014; Henriquez-Henriquez et al., 2015; Koal et al., 2015). In addition, ceramide is metabolized from sphingomyelins and vice versa by sphingomyelinase. Ceramide is known to serve as a secondary messenger for intracellular activation of caspase-3 levels in cellular apoptosis (Haughey et al., 2010). Therefore, combination of changes in ceramide and sphingomyelin levels can predict the overall lipid status of myelin in the brain. Lipids are highly sensitive to changes in brain health, so they offer new diagnostic possibilities due to the development of robust and sensitive analytical methods (Touboul and Gaudin, 2014).

### ^1^H-MRS

MRI is a non-invasive and widely accepted diagnostic modality to study brain abnormalities. While T1, T2, and T2^*^ MRI can provide information related to gross anatomical changes, edema and cerebral hemorrhaging, ^1^H-MRS provides more detailed chemical insight into the functional status and pathological prognosis of the brain (Sajja et al., 2012; Kantarci, 2013). Pre-clinical ^1^H-MRS can resolve ~25 and clinical ^1^H-MRS about ~10 metabolites depending on peak-suppression parameters (Moore and Galloway, 2002; Moffett et al., 2007; Sofroniew and Vinters, 2010).

N-acetyl aspartate (NAA) is a neurometabolite synthesized from aspartate and acetyl co-enzyme A. NAA or NAA/creatine (Cre) is trans-regulated between oligodendrocytes and neurons and can provide insight to structural integrity of WM (Charlton et al., 2006; Ariyannur et al., 2008; Kantarci, 2013). Studies have shown NAA levels in brain correlate with altered WM integrity following TBI (Pendlebury et al., 2000; Brooks et al., 2001; Shiino et al., 2004). Disruption in neuron-oligodendrocyte homeostasis can affect axon potentials and eventually neurotransmission, leading to an altered cognitive status. Since, alterations in the levels of NAA in WM-rich regions could indicate health status of oligodendrocytes and it can be measured by both pre-clinical and clinical ^1^H-MRS, it has the potential to be an innovative translational avenue.

Reactive astrocytes rapidly accumulate in the injury region and alter their morphology, typically inducing swelling. This is related to osmolarity changes that result from edema or ischemia following TBI (Sofroniew and Vinters, 2010). Myo-inositol (Ins) is a primary metabolite that maintains brain osmolarity. Clinical studies have reported that an up-regulation of Ins correlates with astrogliosis in pathophysiological conditions such as TBI, dementia, and glioblastoma (Hattingen et al., 2008; Kantarci, 2013; Kierans et al., 2014). Pre-clinical studies have demonstrated that ^1^H-MRS-resolved Ins was associated with astrogliosis and impaired cognition following TBI (Kierans et al., 2014; Sajja et al., 2014a).

In conjunction with astrocytes, microglia actively participate in clearing debris resulting from neuroinflammation. Changes in metabolites such as phosphoryethanolamine (PEA), glycerophosphocholine (GPC), and cholines (Cho) have been linked to microglia. PEA and GPC are involved in cell membrane turnover indicating neuroinflammation and GPC/PEA levels change depending of cell activation status (Sajja et al., 2012). Thus, they indicate compromised cellular activities.

Resolving ^1^H-MRS peaks with lower signal-to-noise ratios depends on the field strength of the scanner, time of acquisition and number of repetitions of acquisition. Although, many metabolites can be resolved using pre-clinical MR scanners, only a small portion can be resolved with a clinical scanner which limits clinical translation. However, NAA, Ins, and Cho can be resolved with clinically available MR scanners. Thus, we highlighted the potential utility of clinical ^1^H-MRS in combination with other modalities for differential diagnosis.

## Conclusion

We have reviewed several glial-based molecules, that give clues to glia health status following TBI. There is a general consensus that a panel of markers will provide the most clinically relevant diagnostic tool. Thus, understanding how glial dysfunction following injury can alter mechanisms of synaptic plasticity and its relationship to an increased risk for persistent memory deficits is necessary for advancement. Researchers are actively pursuing new targets for a minimally invasive tools which can accurately and objectively detect brain injury. Combining sophisticated tools, such as serum biomarkers and MRS, will provide for an accurate differential diagnosis following TBI. Moreover, a temporal pattern of these markers could offer prognostic clues as to neuronal plasticity leading to memory formations.

## Author contributions

All authors listed, have made substantial, direct and intellectual contribution to the work, and approved it for publication.

### Conflict of interest statement

The authors declare that the research was conducted in the absence of any commercial or financial relationships that could be construed as a potential conflict of interest.
